# Disease of the Nasal Fossæ

**Published:** 1842-10-08

**Authors:** 


*Disease of the Nasal Fossæ.*—This is a case related by Dr. Münchmeyer, of Limeburg. A man, aged 71, suffered at the end of last winter from a severe cold, which subsided under proper treatment. On the 6th of April he was attacked with pain about the left side of the forehead, extending to the left side of the face and the left ear; it was very severe, and deprived him of rest. The left nostril was dry, but nothing abnormal could be seen in it. The left eye was pushed forward, and had an amaurotic appearance, but the sight remained. Various topical remedies to relieve congestion were employed, in spite of which the disease rapidly progressed. The pain increased, the eye was projected beneath the orbit, vision became impaired, and paralysis of the upper eyelid took place. At the same time a small firm swelling appeared at the inner canthus, and acquired the size of a pea. Several severe hæmorrhages occurred from the nose, which reduced the patient to an extreme degree of weakness, and he died May 17, without having experienced any cerebral symptoms.

On examination of the head, a pretty considerable layer of gelatinous substance was found upon the surface of the brain; but it does not seem clear that this had any connection with the other disease. In the left upper nasal cavities was found a growth, resembling in texture a fleshy, very firm polypus, which was situated beneath the cerebriform plate and in the sinuses of the left ethmoid bone, being principally confined to this situation, but constituting also the projection at the inner canthus. The posterior ethmoidal cells and the sphenoidal sinuses were enlarged, and filled with a mass resembling in consistence and appearance thick pus. The mucous membrane lining these cavities was destroyed, and the inner surface of the bone carious to a considerable extent. There had been no escape of the matter towards the interior of the cranium, but it appeared to have gained a partial exit into the nostrils.

The author remarks that, although morbid growths in these cavities are by no means rare, such expansion of the sphenoidal sinuses with destruction of their sinuses are very seldom found. He states that he had been unable to find a similar case recorded in the works of several authors to which he has referred.—*Ibid, from Oppenheim's Zeitschrift für die gesammte Medicin.*

